# Immunological Network in Head and Neck Squamous Cell Carcinoma—A Prognostic Tool Beyond HPV Status

**DOI:** 10.3389/fonc.2020.01701

**Published:** 2020-09-15

**Authors:** Anna Fialová, Vladimír Koucký, Martina Hajdušková, Kamila Hladíková, Radek Špíšek

**Affiliations:** ^1^Sotio, Prague, Czechia; ^2^Department of Otorhinolaryngology and Head and Neck Surgery, First Faculty of Medicine, Charles University and University Hospital Motol, Prague, Czechia; ^3^BioGraphix, Hluboká nad Vltavou, Czechia

**Keywords:** head and neck squamous cell carcinoma, human papillomavirus (HPV), tumor microenvironment, immune infiltrate, antitumor immune response, treatment de-escalation

## Abstract

Head and neck squamous cell carcinoma (HNSCC) is a highly heterogeneous disease that affects more than 800,000 patients worldwide each year. The variability of HNSCC is associated with differences in the carcinogenesis processes that are caused by two major etiological agents, namely, alcohol/tobacco, and human papillomavirus (HPV). Compared to non-virally induced carcinomas, the oropharyngeal tumors associated with HPV infection show markedly better clinical outcomes and are characterized by an immunologically “hot” landscape with high levels of tumor-infiltrating lymphocytes. However, the standard of care remains the same for both HPV-positive and HPV-negative HNSCC. Surprisingly, treatment de-escalation trials have not shown any clinical benefit in patients with HPV-positive tumors to date, most likely due to insufficient patient stratification. The in-depth analysis of the immune response, which places an emphasis on tumor-infiltrating immune cells, is a widely accepted prognostic tool that might significantly improve both the stratification of HNSCC patients in de-escalation trials and the development of novel immunotherapeutic approaches.

## Introduction

Head and neck squamous cell carcinomas (HNSCCs) are a heterogeneous group of epithelial tumors that are localized in the oral cavity, nasopharynx, oropharynx, hypopharynx, and larynx with an estimated global incidence of more than 800,000 new cases per year (1). In general, heavy tobacco and alcohol exposure have been determined to be the most important risk factors for HNSCC. In the 1990s, human papillomavirus (HPV) was described as an emerging etiological agent of oropharyngeal cancer [oropharyngeal squamous cell carcinoma (OPSCC)]. In the following years, the incidence of HPV-associated tumors of the tonsils and base of the tongue has markedly increased, especially in the developed world. Recently, the proportion of patients with HPV-associated OPSCC may be as high as 70–90%, depending on the patients' region of origin (2, 3).

**Figure 1:**
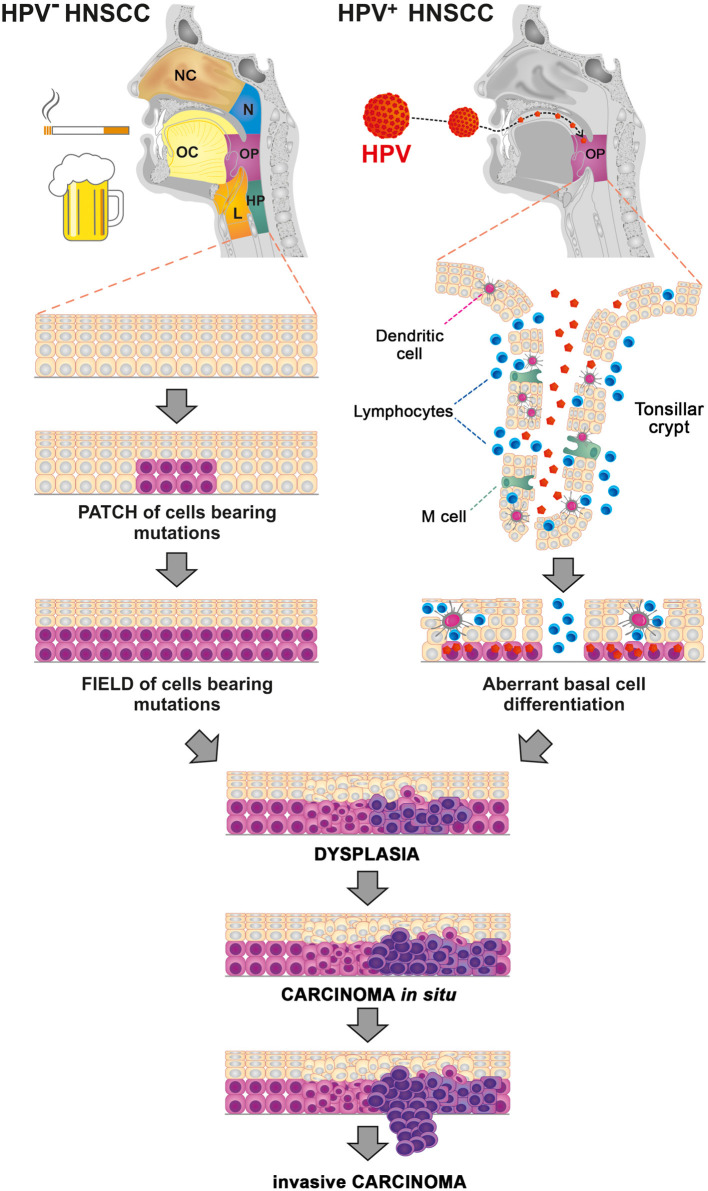
Processes of carcinogenesis in human papillomavirus (HPV)-negative and HPV-associated head and neck squamous cell carcinoma (HNSCC). Tobacco- and alcohol-related mutagens induce widespread mutagenesis in the cells that form the stratified squamous cell epithelium of the upper aerodigestive tract, including the nasal cavity (NC), oral cavity (OC), nasopharynx (N), oropharynx (OP), hypopharynx (HP), and larynx (L). HPV preferentially infects the basal cell layer of the reticulated epithelium of the tonsillar crypts, thus promoting the malignant transformation of epithelial cells within the oropharyngeal region (OP).

HPVs are small double-stranded DNA viruses from the family Papillomaviridae. At present, more than 200 different HPV types have been identified, including 16 “high-risk” types that are preferentially found in precancerous and cancerous lesions (4, 5). In OPSCC, the most commonly detected type is HPV16 (>80%) followed by HPV18 (3%) (6). In contrast to tobacco- and alcohol-related mutagens, which induce mutagenesis in broad areas of the cells that form the stratified squamous cell epithelium of the upper aerodigestive tract, the carcinogenic activity of HPV is localized to the reticulated epithelium of the tonsillar crypts, thereby promoting the malignant transformation of epithelial cells within the oropharyngeal region (Figure 1) (7). Additionally, whereas >80% of HPV-negative tumors bear mutations in *TP53*, HPV-associated tumors mostly harbor wild-type *TP53* (8). During HPV infection, the HPV-derived oncoprotein E6 binds to host tumor suppressor protein p53, inducing its ubiquitin-mediated degradation, whereas the oncoprotein E7 inactivates pRb (9, 10). Inactivation of pRb results in overexpression of p16 (11), which is used as a valid marker for HPV status assessment in OPSCC patients.

Although the process of carcinogenesis differs markedly between HPV-associated OPSCC and HNSCC of other etiology, both types of tumors have a high tumor mutational burden (TMB). In general, tumors with high TMB express higher levels of neoantigens that can be recognized by the immune system (12). Surprisingly, high TMB correlated in HNSCC patients with unfavorable immune expression signatures and poor clinical outcome (13). Besides carcinogen exposure, a significant part of mutations in HNSCC can be attributed to the activity of apolipoprotein B mRNA editing enzyme, a catalytic polypeptide-like 3 (APOBEC3) family of cytosine deaminases. In accordance with the well-defined role of the APOBEC family in viral restriction, APOBEC3 mutations are particularly prominent in HPV-associated OPSCC. Contrary to the general TMB mentioned above, immune cell infiltration was positively associated with APOBEC mutational burden in HNSCC (14, 15).

Smoking and alcohol consumption on the one hand and HPV infection on the other hand can also markedly affect the composition of the salivary microbiome. It has been reported that microbes and their products can influence cancer development and progression, antitumor immune response, and in the upshot patients' survival (16–18). Therefore, the specific impact of the shifts in the oral salivary microbiome during HNSCC progression needs further evaluation.

Patients with HPV-associated tumors are typically diagnosed with large, cystic metastatic cervical lymph nodes; however, they are highly responsive to standard treatment approaches and have significantly better prognoses compared to HPV-negative patients (19–21). Due to the discrepancy between the predictive value of the standard staging algorithm in patients with HPV-negative and HPV-positive HNSCC, the eighth edition of the American Joint Committee on Cancer Staging Manual proposed a new, independent staging system for HPV- associated OPSCC (22). Consequently, since 2018, HPV-associated OPSCC and HPV-negative HNSCC have been considered distinct diseases with independent classification and multiple, significant differences in their clinicopathological features (Table 1). In contrast to squamous cell carcinoma of the oropharynx, the clinical impact of HPV and its detection in non-oropharyngeal HNSCC have not been confirmed to date and need to be further evaluated. *In silico* study published by Chakravarthy et al. (30) showed that although HPV-positive non-oropharyngeal HNSCC shared a gene expression signature and basaloid morphology with HPV-positive OPSCC, HPV-positivity in non-oropharyngeal HNSCC was not associated with improved patients' prognosis. The major difference between HPV-associated non-oropharyngeal and oropharyngeal HNSCC was in the level of tumor-infiltrating immune cells, suggesting a crucial role of immune response in the disease outcome.

**Table 1:** Features of HPV-negative and HPV-positive HNSCC.

**Feature**	**HPV−**	**HPV+**	**References**
Risk factors	Tobacco, Alcohol	HPV	(23)
Incidence	Decreasing	Increasing	(24)
Most common anatomic site	Oral cavity, Larynx	Oropharynx	(20)
Age	Older	Younger	(25)
Race	Non-Caucasian	Caucasian	(20)
Education level	Lower	Higher	(24)
5-years overall survival	48%	80%	(26)
Histological subtype	Keratinizing	Non-keratinizing	(27)
LN metastases	55.7%	86%	(20)
Mutational spectrum	*TP53, CDKN2A, MLL2, CUL3, NSD1, PIK3CA, NOTCH*	*PIK3CA, DDX3X, CYLD, FGFR*	(28)
Density of tumor-infiltrating immune cells	Lower	Higher	(29)

*HNSCC, head and neck squamous cell carcinoma; HPV, human papillomavirus; LN, lymph node*.

The excellent prognosis of HPV-positive OPSCC patients also initiated discussions about treatment de-escalation strategies, which may achieve similar efficacy with decreased toxicity in this particular group of patients (31, 32). The standard treatment regimens, which mainly include curative chemoradiotherapy or surgery followed by adjuvant radiotherapy or chemoradiotherapy, are highly effective; however, they are associated with substantial long-term morbidity, which escalates with treatment intensity and negatively impacts the quality of the patients' lives (32). However, due to the existence of a subgroup of “high-risk” HPV-positive OPSCC patients with a poor prognosis, patient stratification according to HPV status alone is insufficient for successful treatment deintensification. A positive correlation between heavy smoking and poor clinical outcome, as reported by several authors (25, 31, 33), led to the use of smoking status as a cofactor in some de-escalation clinical trials (25, 34). In addition to smoking history, the immune signature might be another important cofactor for the precise selection of patients for de-escalation regimens. Although pan-cancer analyses reveal both HPV-negative and HPV-positive HNSCC as malignancies with a high level of immune cell infiltration (35), HPV-positive OPSCCs show in general markedly higher densities of tumor-infiltrating lymphocytes (TILs) and belong to the immunologically “hottest” of all cancer types (29, 35–37). This feature was reported to be positively correlated with patient survival in a wide range of malignancies (36, 38–42). However, HPV-positive tumors are heterogeneous, and some of the patients with confirmed HPV-associated OPSCC were shown to have immunologically “colder” tumors with low levels of TILs and markedly worse clinical outcome (26, 42, 43). Indeed, Ward et al. (26) described a prognostic model based on the TIL density, smoking status and T stage, and this model can effectively identify the subgroup of HPV-positive patients with poor survival who should be excluded from treatment deintensification trials.

It is widely accepted that the shape of the antitumor immune response is a significant factor that determines a patient's clinical outcome. Thus, it is thought that the detailed characterization of the tumor microenvironment will translate into targeted therapeutic approaches and significant improvements in both overall survival and quality of life following treatment. This review will summarize the knowledge about the immune cell infiltration of the remarkable HNSCC tumor microenvironment with respect to HPV status.

## Immune Microenvironment of Head and Neck Squamous Cell Carcinoma Tumors

**Figure 2:**
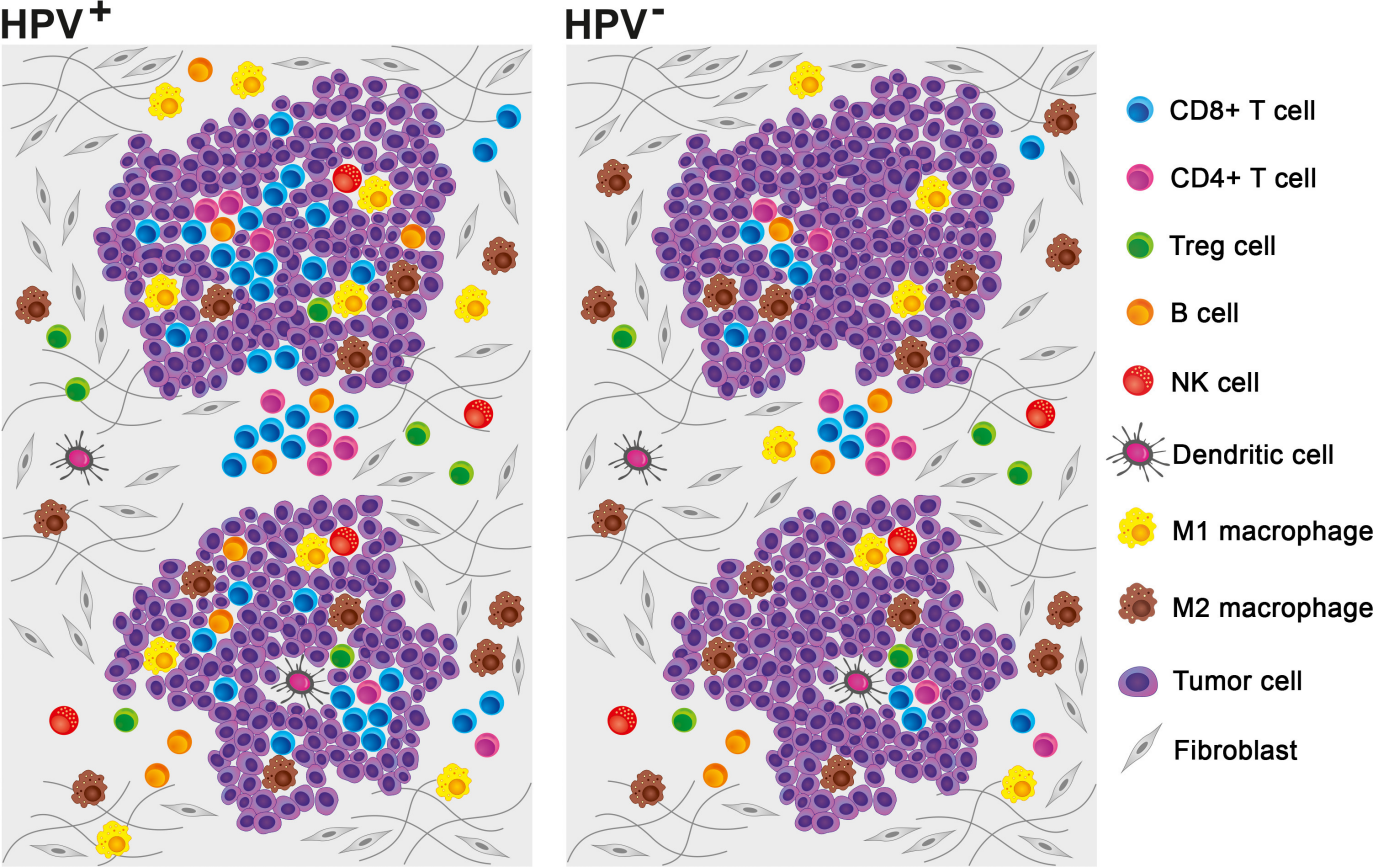
Pattern of tumor-infiltrating immune cells in the human papillomavirus (HPV)-negative and HPV-associated head and neck squamous cell carcinoma (HNSCC) microenvironments. Although myeloid cells prevail in the HPV-negative tumor microenvironment, HPV-associated tumors are mostly characterized by high numbers of tumor-infiltrating lymphocytes. Neutrophils and myeloid-derived suppressor cells (MDSCs) are not included, as it was not possible to extrapolate the relative proportions of these cell populations from published data.

In the 1950s, the theory of immune surveillance was proposed by Burnet (44). According to this concept, the immune system constantly recognizes and destroys emerging malignant cells before they can develop into detectable tumors. This theory is supported by the fact that cancers, including HNSCC, are more prevalent in immunosuppressed patients (45, 46). To escape the control of the immune system, tumor cells develop multiple strategies that make them unrecognizable by immune cells or that efficiently suppress the immune response. The mechanisms of tumor immune evasion include the reduction of antigen presentation due to the loss of major histocompatibility complex (MHC) class I expression, the production of immunosuppressive cytokines, such as interleukin (IL)-10 and transforming growth factor (TGF)-β, the resistance to apoptosis, and the expression of Fas ligand (FasL), which is capable of inducing the death of TILs (47). Together with the recruitment of regulatory T cells (Tregs) and myeloid-derived suppressor cells (MDSCs) into the tumor, these mechanisms help to establish an immunosuppressive microenvironment, which supports tumor growth (47, 48). Despite the prevailing immunosuppressive character, the pattern of immune cell infiltrate markedly differs between HPV-associated and HPV-negative tumors (29, 37) (Figure 2). Indeed, not only the density of tumor-infiltrating immune cells but also their phenotypes and functional capacities distinguish immunologically “hottest” HPV-positive tumors with good prognosis from immunologically “colder,” high-risk HNSCC. The individual features of the tumor-infiltrating immune cell populations are discussed below, and their prognostic impact is summarized in Table 2.

**Table 2:** Prognostic impact of tumor-infiltrating immune cell populations in HNSCC.

**Prognostic marker**	**Impact on prognosis HPV-**	**Impact on prognosis HPV+**	**References**
High M1/M2 ratio	Positive	Positive	(49)
MDSC	NA	NA	
Neutrophils	None	Negative	(49)
NK cells	Positive	NA	(50)
mDC	Positive	None	(51–54)
pDC	Negative	NA	(55, 56)
CD8+ T cells	Positive	Positive	(36, 42, 43, 57)
CD4+ T cells	None	None	(49, 57, 58)
Tregs	Contradictory	Contradictory	(59–63)
B cells	NA	Positive	(43)
IL-10+ Bregs	Negative	NA	(64)

*HNSCC, head and neck squamous cell carcinoma; HPV, human papillomavirus; M1/M2 ratio, ratio between inflammatory M1 and anti-inflammatory M2 macrophages; NK cells, natural killer cells; MDSC, myeloid-derived suppressor cells; mDC, myeloid dendritic cells; pDC, plasmacytoid dendritic cells; Bregs, regulatory B cells; NA, not available*.

## Tumor-Associated Macrophages

Macrophages are monocyte-derived innate immune cells that, as sentinel and effector cells, play an essential role in the maintenance of tissue homeostasis, the control of pathogens, and the overall surveillance of tissue changes (65). According to their mechanisms of activation and subsequent roles in the polarization of the immune response, macrophages are divided into two main phenotypes. Inflammatory “fighting” M1 macrophages are activated by interferon (IFN)-γ and are involved in antitumoral helper T (Th)1 immune responses. Anti-inflammatory “healing” M2 macrophages, which are alternatively activated by IL-4, IL-10, IL-13, and/or prostaglandin E2, are associated with protumoral Th2 immune responses (66–69).

Macrophages are mainly recruited from the bone marrow *via* colony-stimulating factor 1 (CSF-1) and monocyte chemotactic protein 1 (MCP-1) signaling, which are particularly driven by the hypoxic conditions in the tumor tissue (70, 71). M1 macrophages express inducible nitric oxide synthase (iNOS) and produce nitric oxide (NO), IL-12, IL-23, tumor necrosis factor (TNF), IL-1β and IL-6, whereas anti-inflammatory M2 tumor-associated macrophages (TAMs) secrete immunosuppressive cytokines and express arginase-1, which promotes the depletion of extracellular arginine and leads to the metabolic suppression of tumor-infiltrating T cells (65, 69, 71). Additionally, TAMs, as a major source of C-C motif chemokine ligand (CCL)22, help recruit Tregs into the tumor microenvironment *via* the CCL22/C-X-C motif ligand (CXCL)4 pathway (72, 73).

In HNSCC, TAMs generally show the tumor-promoting M2 phenotype that is associated with the production of the immunosuppressive cytokines IL-10 and TGF-β, and their presence in the tumor microenvironment is positively correlated with lymph node status and poor prognosis (71, 74–76). However, although the overall density of TAMs is comparable in HPV-positive and HPV-negative tumors (29, 37), Gameiro et al. (37) reported a significantly lower proportion of M2 macrophages in HPV-associated tumor tissues compared to that in HPV-negative tumor tissues. Similarly, Chen et al. (49) observed a higher M1/M2 macrophage ratio in HPV-positive tumors compared to that in HPV-negative tumors. Importantly, a high M1/M2 ratio correlated with better prognosis in both HPV-positive and HPV-negative HNSCC patients. Both analyses were performed at the mRNA level using publicly available databases.

## Myeloid-Derived Suppressor Cells

Myeloid-derived suppressor cells (MDSCs) form a heterogeneous population of immature myeloid cells, which under physiological conditions represent only 0.5% of peripheral blood mononuclear cells (PBMCs) and consist of precursors of granulocytes, monocytes, and dendritic cells. There are two major subsets of MDSCs in humans, namely, Lin^−^HLA-DR^−/lo^CD11b^+^CD14^−^CD15^+^CD33^+^ granulocytic PMN-MDSCs and Lin^−^HLA-DR^neg/lo^CD11b^+^CD14^+^CD15^−^ monocytic M-MDSCs (77, 78). Pathological MDSC accumulation is associated with chronic inflammation and cancer progression, and MDSCs are known to exhibit significant immunosuppressive and protumorigenic functions. These tumor-promoting activities include the production of immunosuppressive cytokines IL-10 and TGF-β, the secretion of angiogenic factors, NO and reactive oxygen species (ROS), the promotion and activation of Tregs, and the induction of arginine and cysteine deprivation, which result in the metabolic suppression of tumor-infiltrating T cells and the production of soluble factors that support tumor growth and invasion (71, 77, 79, 80).

MDSCs are mainly recruited to the tumor microenvironment *via* the prostaglandin E2-induced chemokines CCL2, IL-8, and CXCL12 (80, 81). Additionally, tumor cells are capable of producing mediators of chronic inflammation, such as granulocyte-macrophage colony-stimulating factor (GM-CSF), vascular endothelial growth factor (VEGF), TNF-α, IL-1β, and IL-6, which induce the generation and expansion of MDSCs *in situ* (80, 82). In HNSCC patients without a defined HPV status, the proportion of circulating PMN-MDSCs negatively correlated with overall survival. These peripheral blood-derived MDSCs were capable of suppressing T cell proliferation and cytokine production (83). Similarly, Chikamatsu et al. (84) reported elevated levels and suppressive activities of MDSCs in the peripheral blood of HNSCC patients. In HPV-negative HNSCCs, tumor-derived MDSCs created a significant proportion of tumor-infiltrating immune cells and were capable of efficiently suppressing T cell (85) and natural killer (NK) cell functions (86). As all of these studies either did not specify the HPV status of the patients or included HNSCC patients with tumors localized outside the oropharynx, there is no report about the proportions and suppressive capacities of MDSCs in HPV-associated HNSCC to date.

## Neutrophils

Neutrophils represent the most abundant population of immune cells in humans and play an essential role in antimicrobial immune responses and wound healing (87). Depending on the signals from the tumor microenvironment, neutrophils can be either protumorigenic or antitumorigenic; however, most published studies describe neutrophils as tumor-promoting cells with a strong impact on the antitumor immune response (87, 88).

Similar to other malignancies, neutrophils were found at elevated levels in the peripheral blood of HNSCC patients, and their frequencies were inversely correlated with the frequencies of lymphocytes (89, 90). Patients with HPV-associated tumors had significantly lower levels of circulating neutrophils compared to patients with HPV-negative tumors, and the high absolute number of neutrophils correlated with poor prognosis in HPV-positive patients but not HPV-negative patients (91). However, if the abundance of neutrophils was related to the levels of circulating lymphocytes, a high neutrophil-to-lymphocyte ratio (NLR) was associated with poor prognosis in both groups of patients. As expected, patients with HPV-associated tumors showed lower NLR ratios compared to patients with HPV-negative tumors (89). Surprisingly, in patients with advanced oral squamous cell carcinoma (OSCC), both very high and very low NLRs were reported to be associated with increased risk of death (92). The authors suggest that compared to early-stage OSCC, where low NLR indicates unaffected immune system, in advanced-stage tumors, very low NLR may be a marker of immune system exhaustion.

The only publication that mentions the levels of neutrophils in the tumor microenvironment is an *in silico* study published by Chen et al. (49), which reported significantly lower levels of tumor-infiltrating neutrophils in HPV-associated samples compared to those in HPV-negative samples. Additionally, high infiltration of neutrophils was correlated with poor outcome in patients with HPV-associated HNSCC and was determined to be an independent prognostic marker based on the Cox proportional hazard model.

## Natural Killer Cells

NK cells are generally considered to be effector lymphocytes of the innate immune system; however, they express a wide spectrum of activating and inhibitory receptors, which efficiently empower their cytotoxicity against virus-infected and tumor cells while concurrently ensuring self-tolerance (93). NK cells are known to recognize cells that escape detection by cytotoxic T cells due to the abnormal surface expression of HLA class I molecules. Indeed, a reduction in HLA class I expression is a very common mechanism used by viruses, such as HPV, and tumor cells to evade the host immune response (94). There are two major groups of NK cells, namely, cytokine-producing CD56^bright^CD16^dim^ immunoregulatory NK cells and CD56^dim^CD16^bright^ cytotoxic NK cells.

In HNSCC patients, peripheral CD56^dim^ NK cells were shown to be functionally impaired and preferentially targeted for apoptosis (95). Subsequently, plasma TGFβ1 and soluble MHC class I chain-related peptide A (sMICA) were determined to be the main factors driving the loss of the functional capacities of peripheral NK cells in HNSCC (96). Although an *in silico* study published by Chen et al. (49) revealed no difference between the NK cell gene signatures in HPV-negative and HPV-positive HNSCC samples, Wagner et al. (50) found significantly higher numbers of tumor-infiltrating CD56+ NK cells in the microenvironment of HPV-positive OPSCC specimens compared to those in the microenvironment of HPV-negative OPSCC specimens. These cells mostly coexpressed granzyme B and CD16, suggesting their cytotoxic capacity and were correlated with increased overall survival independent of the HPV status of the patients.

## Myeloid Dendritic Cells

Myeloid dendritic cells (mDCs) are the most important antigen-presenting cells (APCs) with the highest capacity to initiate adaptive immune responses. Immature mDCs efficiently capture and process antigens, but due to the lack of co-stimulatory molecules, they are rather tolerogenic and may actually inhibit T cell responses (97, 98). Upon stimulation with microbial stimuli and inflammatory cytokines IL-1, TNFα, and IL-12, mDCs undergo maturation and migrate into T cell-rich areas of lymphoid organs. Mature mDCs produce substantial amounts of IL-12 and express high levels of HLA molecules and high levels of co-stimulatory molecules that are equally essential for T cell activation (99, 100).

Compared to healthy controls, HNSCC patients had significantly lower numbers of CD11c+ DCs in their peripheral blood. Interestingly, the decreased mDC levels normalized after tumor resection (101). In squamous cell carcinoma of the tongue, the presence of a high level of peritumoral CD1a+ DCs was shown to be associated with improved overall patient survival (52). High densities of stromal CD1a+ Langerhans cells were later confirmed to be a positive prognostic marker in HPV- HNSCC but not in HPV+ HNSCC (54). Similarly, in laryngeal (51) and oral (53) cancer patients, low densities of S-100+ DCs were associated with poor prognosis. To the best of our knowledge, compared to HPV-negative HNSCC, mDCs have not been considered a valid prognostic factor in HPV-associated oropharyngeal tumors to date.

*In silico* studies published by Chen et al. (49) and Gameiro et al. (37) did not reveal any statistically significant differences in the expression of mDC-related genes between HPV-positive and HPV-negative HNSCC samples (37, 49). In contrast, we observed significantly higher levels of CD45+LIN-HLA-DR+CD14-CD11c+ mDCs in HPV+ oropharyngeal tumors compared to those in HPV-negative HNSCC using flow cytometry (29). However, we did not show any differences between the densities of tumor-infiltrating DC-LAMP+ activated mDCs in HPV+ and HPV- oropharyngeal tumor samples using immunohistochemical staining, and we did not observe any associations between the DC-LAMP+ mDC densities and patient outcomes (43).

## Plasmacytoid Dendritic Cells

Plasmacytoid dendritic cells (pDCs) play an essential role in the antiviral immune response and are characterized by their considerable production of IFNα in response to viral RNA or DNA, which are recognized by intracellular Toll-like receptors TLR7 and TLR9, respectively (102). Additionally, depending on the activation status of pDCs, these cells may act as efficient antigen-presenting cells or induce the differentiation and expansion of Tregs (103, 104).

Similar to other solid tumors, the pDCs infiltrating HNSCC were shown to be functionally impaired and were thought to be rather protumorigenic. Indeed, Hartmann et al. (105) reported a diminished capacity of HNSCC-infiltrating pDCs to produce IFNα upon TLR9 stimulation with CpG motif-containing oligonucleotides. Moreover, tumor-derived supernatants harvested from primary tumor cell cultures and HNSCC cell lines inhibited IFNα production in control peripheral pDCs. Bruchhage et al. (106) later suggested that IL-10 might be the major cytokine responsible for the impairment of pDC functional capacity in the HNSCC microenvironment. Consistent with these findings, high densities of pDCs were associated with poor prognosis in oral squamous cell carcinoma patients (55, 56).

## T Lymphocytes (Tumor-Infiltrating Lymphocytes)

T lymphocytes are the pillars of adaptive immunity and are known to be essential in the control of tumor progression. Consequently, most of the immunotherapeutic protocols in cancer management, including highly successful immune checkpoint inhibitors, target T cell-related immune responses. Three major classes of T cells can be distinguished according to their primary function: cytotoxic CD8+ T cells, which are capable of killing infected or malignant cells; helper CD4+ T cells, which provide essential signals to B cells and polarize the immune response *via* cytokine production; and Tregs, which suppress the activity of other lymphocytes and help maintain peripheral tolerance.

Similar to the observations in other malignancies, the densities of CD8+ tumor infiltrating T cells were positively correlated with improved clinical outcome in both HPV-associated and HPV-negative HNSCC (36, 42, 43, 57, 107, 108). In general, tumors associated with HPV show significantly higher levels of T cell infiltration, especially CD8+ T cell infiltration (29, 36, 37, 49). Additionally, significantly higher proportions of CD8+ T cells infiltrating HPV-associated HNSCC were reported to be capable of producing pro-inflammatory cytokines, namely, IFNγ and IL-17 (29). However, a subgroup of cases with low proportions of infiltrating TILs and prognosis comparable to that of patients with HPV-negative tumors can be identified among HNSCC patients with HPV-positive tumors (26). These data suggest that the quantity and quality of the immune infiltrate is a valid prognostic tool that may markedly improve the stratification of HNSCC patients. Indeed, it has been shown that HPV-specific CD8+ T cells are detectable in 64–75% of HPV-positive HNSCC samples (109–111). These functional HPV-specific T cells were shown to be mostly PD-1+Tim-3- (111), and their presence was associated with improved overall survival (110). Thus, in addition to the density of CD8+ T cells, the presence of HPV-specific T cells seems to be a valid prognostic marker that can be used for better patient stratification.

In the case of CD4+ T cells, our study based on flow cytometry data showed significantly higher numbers of naive CD4+ T cells but not Th1 cells and Th17 cells in the tumor microenvironment of HPV-positive HNSCC samples compared to those in the tumor microenvironment of HPV-negative samples (29). A gene expression study published by Gameiro et al. (37) revealed higher numbers of follicular T helper (Tfh) cells and Tregs, but not memory CD4+ T cells, in HPV-associated tumor samples compared to those in HPV-negative tumor samples. Higher numbers of Tregs in HPV-positive HNSCC were also reported by several studies based on immunohistochemical staining of tumor sections (36, 58, 112). Unlike CD8+ T cells, the role of Tregs in HNSCC is not fully understood. Whereas, some studies suggest a negative impact of tumor-infiltrating Tregs on disease progression (60, 62), other publications reported a positive correlation between high densities of Tregs and patient outcome (59, 61, 63). The high numbers of tumor-infiltrating Tregs observed in immunologically “hot” HPV-associated tumors suggest that the proportions of Tregs or the CD8+ T cell/Treg ratio, rather than Treg numbers alone, might truly reflect the shape of the immune response within the tumor microenvironment. Indeed, we have observed that although the numbers of Tregs were slightly higher in HPV-associated HNSCC samples, the proportions of these cells were actually lower (29). Thus, the whole pattern of immune cells, which also reflects the relationships among various cell populations, provides the best information about the prevailing status of the immune response within the tumor microenvironment.

## B Lymphocytes

It is well-known that B lymphocytes play a central role in humoral immunity due to their capacity to produce antibodies. Different subsets of B cells are able to recognize either polysaccharides or lipid antigens, which leads to T cell-independent responses, or protein antigens, which are presented to Tfh cells in the lymph nodes, Payer's patches, and spleen *via* HLA class II molecules. During T cell-dependent activation, Tfh cells stimulate B cell activation and differentiation into antibody-secreting plasmablasts *via* the CD40L-CD40 pathway and IL-21 and IL-4 production. Additionally, B cells can undergo further maturation in germinal centers and develop either into long-lived plasma cells that secrete high levels of antibodies or into memory B cells. Compared to the T cell-independent pathway of B cell activation, the T cell-dependent pathway of B cell activation leads to the production of high affinity class-switched antibodies (113, 114). In addition to antibody production, B cells are capable of producing immunomodulatory cytokines and chemokines, can play a role as antigen-presenting cells, and can efficiently stimulate both CD4+ T cells and CD8+ T cells (114–116).

Compared to T cells, the role of B cells in cancer immunology has been less extensively explored and generally underestimated. Thus, the role of B cells in tumor progression remains controversial. Whereas, B cells were shown to be rather protumorigenic in mice, high levels of tumor-infiltrating B cells in humans were mainly associated with good outcome and longer overall survival (114, 117). However, recent studies have shown that B cells play an essential role in the response to immune checkpoint inhibitors and thus might be much more important for successful immunotherapeutic approaches than expected (118).

In HNSCC, B cell signatures were able to distinguish between HPV-associated and HPV-negative carcinomas, with a significantly higher expression of B cell-related genes in HPV-associated tumors (37, 43, 49, 119). These data were confirmed at the cellular level, and significantly higher densities of tumor-infiltrating CD20+ B cells were observed in the microenvironment of HPV-associated tumor sections than in the microenvironment of HPV-negative samples (43, 112, 120). Compared to samples with low infiltrates of lymphocytes, B cells derived from TIL-rich tumors were shown to be activated and to express high levels of HLA and costimulatory molecules. Consistent with these findings, high B cell density was associated with good prognosis in OPSCC patients regardless of HPV status (43). Importantly, B cells were shown to create aggregates with CD8+ T cells, and the frequency of these B cell–CD8+ T cell interactions was positively associated with the proportions of HPV-specific CD8+ T cells infiltrating the tumor microenvironment, suggesting the importance of B cells for the T cell-related antitumor immune response (43). In contrast, the proportion of IL-10-producing regulatory B cells (Bregs) in HPV-associated tumor tissues was comparable to the levels of Bregs in control tonsils, indicating that Bregs do not accumulate in the tumor microenvironment of HPV-associated HNSCC (43). In HPV-negative tongue squamous cell carcinoma, the proportions of IL-10+CD19+ Bregs were also very low (below 1%); however, their levels were significantly enhanced compared to adjacent tissue and were significantly correlated with poor outcome in univariant, but not multivariant, survival analysis (64).

Besides the direct association between B cell densities in the tumor microenvironment and the disease outcome, the presence of antibodies against HPV16 E6 and E7 oncoproteins in patients' sera was positively correlated with the recurrence-free survival of HPV-positive OPSCC patients (121, 122). These findings support the importance of B cell-mediated immune responses in HPV-associated OPSCC.

## Cytokine and Chemokine Profile

Similar to other malignancies, higher levels of pro-angiogenic cytokines IL-8 and VEGF were detected in HNSCC patients' sera compared to healthy controls (123). Expression of these cytokines by HNSCC cells was confirmed by immunohistochemistry (IHC), showing up to 90% of VEGF-positive tumors (123, 124). Together with pro-angiogenic effects, IL-8 and VEGF are known to promote tumor growth and metastasis (125). Comparing plasma levels of cytokines in HNSCC patients and healthy controls, Lathers et al. (126) showed that the cytokine profile of HNSCC patients is shifted toward Th2 bias. Indeed, HNSCC patients had significantly higher levels of IL-4, IL-6, and IL-10 in the plasma compared to controls. In agreement with this finding, lower levels of IFNγ were observed in HNSCC patients; however, the levels of IL-1, IL-2, and GM-CSF were increased, whereas Th1 cytokine IL-12 and immunosuppressive TGFβ remained unchanged (126). IL-6 and IL-10 were detected in HNSCC cell lines, primary HNSCC cells, as well as tumor-infiltrating immune cells (123, 127–129). Moreover, serum levels of IL-6 negatively correlated with HNSCC patients' prognosis (130). Despite exerting many pro-inflammatory properties, protumorigenic IL-6 is a pleiotropic cytokine, which affects cell growth, maturation, survival, and migration during immune responses (131, 132). In colorectal cancer, IL-6 was shown to stimulate IL-10 production by tumor cells (133). The role of IL-10 in cancer progression has been extensively studied. Mostly, IL-10 is regarded as an immunosuppressive, anti-inflammatory cytokine, which promotes tumor escape from immune surveillance. However, IL-10 was also shown to inhibit tumor-induced angiogenesis, enhance the production of NO, and increase tumor cell line immunogenicity in some preclinical models (134). Besides pro-angiogenic and Th2 cytokines, HNSCC tissues were reported to produce high levels of pro-inflammatory TNFα (29, 127). Immunohistochemical staining revealed that TNFα is mainly produced by tumor cells, TAMs, endothelial cells, stromal fibroblasts, and inflammatory tumor-infiltrating immune cells (127, 135, 136).

As most of the studies did not include HPV status, little is known about the differences in cytokine profile of HPV-positive and HPV-negative HNSCC. Partlová et al. (29) reported no statistically significant differences in cytokine production in cell culture supernatants derived from HPV-positive and HPV-negative HNSCC, although HPV-positive samples produced higher levels of IL-2, IL-17, IL-23, and IFNγ and slightly lower levels of IL-1β, IL-6, and TNFα compared to HPV-negative samples. However, HPV-positive samples produced markedly higher levels of pro-inflammatory chemokines CXCL9 and CXCL10, which characterize immunologically “hot” tumors (137). Additionally, HPV-positive samples produced significantly higher levels of CCL17 and CCL21. *Via* interaction with CCR4 and CCR8, CCL17 induces chemoattraction of T cells (mainly Tregs and Th2 cells), macrophages, and activated NK cells (138–140). Surprisingly, in HNSCC, the levels of CCL17 positively correlated with the densities of Th17, Th1, and cytotoxic T cells, but not Tregs and macrophages (29). In secondary lymphoid organs, CCL21 attracts naive T cells facilitating their co-localization with antigen-stimulated DCs in T cell zones. In addition to chemoattraction, CCL21 favors expansion of CD4^+^ and CD8^+^ T cells and induces Th1 polarization, whereas Tregs are hyporesponsive to both CCL21-induced migration and CCL21 co-stimulation (141). In HNSCC, levels of CCL21 positively correlated with the frequency of Th17 cells (29).

## Conclusions

Despite the markedly better prognosis of HNSCC patients with HPV-associated tumors and despite the recent segregation of HPV-associated and HPV-negative HNSCC into two different entities, the standard of care management remains the same in both groups of patients. Clinical trials focused on treatment deintensification strategies have not provided the necessary evidence to date to support deintensification protocols. The recently published multicenter DeESCAlaTE and RTOG 1016 clinical trials showed a significant decrease in tumor control in patients with HPV-associated OPSCC treated with radiotherapy plus cetuximab compared to those treated with radiotherapy plus cisplatin-based chemotherapy, and, moreover, there was no benefit in terms of reduced toxicity (142, 143). Indeed, the appropriate selection of patients who would profit from deintensified treatment is essential; however, a valid biomarker that is suitable for the precise stratification of patients with HPV-associated tumors has not yet been approved. As the density and pattern of the immune infiltrate in tumor tissues has been repeatedly associated with patient outcome in a wide range of malignancies, including HPV-associated HNSCC, high densities of CD8+ T cells and especially B cells or the presence of HPV-specific T cells within the tumor tissue might be considered possible biomarkers in treatment deintensification clinical trials. However, these markers would be applicable in surgically treated patients only, as tissue specimens are necessary for precise IHC or flow cytometry-based analyses. For non-surgically treated patients, IL-6 plasma levels and NLR might be candidates for stratification biomarkers. Nevertheless, to validate a biomarker, a large multicenter study needs to be performed to establish a proper cutoff. A precise and comprehensive immune monitoring of completed deintensification clinical trials would enable to preselect a biomarker worth validating.

The current knowledge about the HNSCC microenvironment might be also translated into novel immunotherapeutic approaches. Immune checkpoint inhibitors (ICIs) made a true breakthrough in cancer immunotherapy; nevertheless, primary or acquired resistance often accompanies this approach. Strategies combining multiple approaches thus achieve the highest response rate in cancer patients. In HNSCC, anti-PD-1 monoclonal antibodies nivolumab and pembrolizumab were recently approved as first-line treatment for patients with metastatic or unresectable, recurrent disease (144). Enhancement of Tim-3 expression on T cells following PD-1 blockade as a mechanism of acquired resistance (145) provides a rationale to combine anti-PD-1 therapy with anti-Tim-3 antibodies. High efficacy of simultaneously administered antigen and anti-PD-1 antibody (146) and the absence of Tim-3 overexpression in HPV E6/E7 peptide-stimulated T cells following PD-1 blockade (111) favors combining immune checkpoint inhibitors with HPV-specific vaccine. Indeed, the overall response rate of 33% was achieved with this approach in a phase 2 clinical trial enrolling incurable HPV16-positive OPSCC patients (147).

The importance of B cells in both patient stratification (43) and response to anti-PD-1 therapy (118, 148) suggests that B cells might be a useful target in future immunotherapy protocols. Thus, B cell-activating molecules, such as CD40 agonist antibodies, which are already tested in multiple clinical trials (149), might be interesting partners in novel combination approaches to immunotherapy.

Consequently, patient stratification as well as present immunotherapeutic approaches might be further refined based on the current knowledge of the HNSCC microenvironment, allowing beneficial changes in the standard of care for the treatment of HPV-associated HNSCC.

## Author Contributions

AF and VK wrote the manuscript. MH prepared the illustrations. KH searched for publications in public databases. RŠ reviewed the manuscript. All authors contributed to the article and approved the submitted version.

## Conflict of Interest

AF, VK, KH, and RŠ are employed by the company Sotio, a biotechnological company developing innovative cancer therapies. MH is employed by the company BioGraphix, a company providing scientific illustrations.
